# Plural Pregnancy, Involving a Question as to Sire1Read before the Virginia State Veterinary Medical Association, June 18, 1895,

**Published:** 1895-10

**Authors:** W. H. Harbaugh

**Affiliations:** Richmond, Va.


### PLURAL PREGNANCY, INVOLVING A QUESTION AS TO SIRE.[1]

BY DR. W. H. HARBAUGH,
RICHMOND, VA.

In the course of our practice we are often asked a question difficult to answer, but the following is of more than usual interest, not only on account of the question involved, but because it carries us into the ever-romantic domain of obstetrical physiology. Many as are the ascertained facts in connection with the function of reproduction, there probably remains more theory and more speculation in regard to it than any other function of the animal economy.

[1] Read before the Virginia State Veterinary Medical Association, June 18, 1895.

The facts of the case are as follows: A brown mare, five years old, a primipara, by Woodburn Hambletonian, was first served by Norfolk, November 22, 1888, but failed to get with foal. She was served again by Norfolk, March 1, April 1 and 10. She was in heat again and was served by Eggwood on April 30. She was changed to the last-named stallion because her owner thought the first-named could not get her with foal. She did not exhibit any signs of heat after Eggwood served her, and her owner considered her in foal to Eggwood. At about what they said would have been near the regular time for her to foal to Norfolk (the exact date I could not ascertain), she gave birth to twins; one was a chestnut, resembling Norfolk in every respect, well matured, born alive, but died within a day after birth; the other was a bay resembling Eggwood, born dead, and showing plainly that he was foaled before maturity.

These particulars were given me, and I was requested to give an opinion as to which of the stallions should be credited with getting the mare in foal. I fully realized the importance of the question, and in order to impress upon you the importance of it it is only necessary to remind you that it is a common custom in our State for stallion-owners to guarantee to get a mare in foal, consequently the fee for stallion-service becomes involved and the case is liable to be carried to court.

My opinion was that this was a case of superfecundation: that the chestnut stallion, Norfolk, was the sire of the chestnut foal, and that the bay stallion, Eggwood, was the sire of the immature bay foal.

There should be reason in all things, and it will not be out of place to give reasons for the opinion expressed, but in doing so I will have to resort to theory, speculation, and analogy, as well as facts pertaining to the various phenomena connected with reproduction.

Heat, or the period of œstrum, in the mare is analogous to menstruation in women, but in the latter the periods are more regular as a rule than in the mare. In the mare there is also much irregularity as to the duration of heat; in some it lasts but a day or two, in others it persists for five or six days, or even longer. As regards the length of time between the periods there exist many different opinions; some observers say the periods of œstrum occur every eighteen days, others say every twenty or twenty-one days, and still others say every nine days. Stallion-owners in my section of the State request that mares be returned for "trial" every nine days. While we must admit that experience points to the fact that the mare is most sure to conceive if put to the stallion on the ninth day after parturition, it by no means follows that the period of œstrum occurs every nine days. After close observation of my own mares for years, I can set no exact time for the recurrence of the periods, and I must say they are very irregular, although I think every three weeks during spring and summer comes nearer being the rule than the other periods mentioned. In some mares the period of œstrum is not accompanied by any signs manifest to ordinary observation, and in such mares it can only be known by "trying" them with a stallion.

The period of œstrum corresponds with certain phenomena in the ovary; at each period a Graafian vesicle is matured, from which is discharged an ovum. The mare being a uniparous animal only one ovum escapes, as a rule, at each period. When the Graafian vesicle is about ready to burst, that part of the ovary where the vesicle matured is embraced by the fimbriated extremity of the Fallopian tube, and the ovum finds its way into the tube, and thence into the cavity of the uterus.

The spermatozoa, the fertilizing element of the male, are ejected into the vagina of the mare during the act of copulation, and they find their way into the uterus and up the Fallopian tubes, and when they come into contact with the ovum fecundation results. Some assert that the ovum is fertilized in the uterus, while others think that it is fertilized even before it escapes from the Graafian vesicle, but the majority of observers are of opinion that the ovum is impregnated while in the Fallopian tube on its way to the uterus. There can be no doubt that the spermatozoa ascend the Fallopian tubes, which is fully proved by the instances of extra-uterine pregnancy, such as the tubal, ovarian, and abdominal pregnancies, and also by the positive fact of finding them there in a limited time after coition, as well as by finding the fecundated ovum in the tube. In some animals spermatozoa have even been discovered on the surface of the ovary in a comparatively short time after coition.

Then, after the ovum is fecundated, it descends into the uterus, where it becomes attached to the mucous membrane and undergoes the developments which ultimately result in an independent being.

The envelopes, as well as the embryo, in the mare are formed from the ovum; the outer enveloping membrane is attached to the internal surface—the mucous membrane—of the uterus by the placenta. This external envelope is called the chorion. Within it is the allantois, one part adherent to the internal surface of the chorion, the other part reflected against and loosely adherent to the external surface of the amnion, somewhat after the manner of the relation of the pleura to the surface of the lung and the internal wall of the thoracic cavity. The amnion is the envelope that contains the fluid in which the fœtus is suspended. In the human female there is another membrane called the decidua, which envelops the chorion and is formed by a modification of the mucous membrane of the uterus, which I will illustrate on the blackboard presently. There are other differences in the human fœtal envelopes not necessary to refer to here.

When the fecundated ovum reaches the uterus, it must be remembered that it is not attached to the mucous membrane immediately by the placenta, as they are not formed till a later period. We may say, however, for want of a better expression, that the ovum becomes grafted to the mucous membrane and is surrounded by a mass of albuminoid substance from which it derives nourishment. As the ovum develops and enlarges the changes gradually take place, and the placenta of the chorion are formed and penetrate into corresponding depressions in the uterine mucous membrane where the changes in the fœtal blood occur.

The foregoing review but briefly refers to the usual phenomena in the mare. Now we will take up the subject of twins.

Leishman goes into the subject of plural pregnancy in an exhaustive manner and reviews the different ways in which twins occur. In this article, however, I must be brief.

A single ovum may contain two yolks. Two ova may exist in a single Graafian vessel which may escape and be impregnated together, or successively. Two ova may form within two Graafian vesicles in the same ovary, or one in each ovary, the latter being proved by the simultaneous occurrence of pregnancy in each cavity of a double uterus, and by the existence of two corpora lutea in the same stage of development.

Most cases of twins occur when two distinct ova are impregnated, whether from separate ovaries or from two Graafian vesicles in the same ovary, or from a single Graafian vesicle. Each of these becomes imbedded in the mucous membrane of the uterus, and the decidua reflex arises round it in the usual way. In the process of growth the two tumors approach each other and come into contact, the partition between the two fœtal cavities consisting of the membranes of each fœtus respectively. As a rule, there is no vascular communication between them. Another and very rare class of cases is where there is a chorion common to both embryos, but each inclosed in its own amnion. This is presumed to be the result of the impregnation of two germs within a single ovum—a double yolk. It is also said that two embryos occasionally exist in a single amniotic cavity, which is supposed to be of the same class as the last, only that the amniotic partition has been absorbed.

Fecundation, conception, impregnation, are terms applied, meaning that the ovum has been fertilized by the male element, the spermatozoa.

Superfecundation implies that more than one ovum has been fecundated or fertilized in the same female, at one time or successively, during the same pregnancy; that is to say, that the impregnation of one ovum is succeeded by the impregnation of another ovum in the same female within a limited time after the first impregnation.

Superfœtation is often used to convey the same meaning as superfecundation, but in this article I will restrict the meaning of the term to such cases as may seem to support the assertion that a second fecundation has taken place after a considerable interval, but during the same pregnancy.

The opinion prevails that shortly after conception the uterus becomes hermetically sealed, and so remains till the end of gestation. The sealing of the uterus is effected, first, by a mucous plug in the cervix, and, secondly, by the fœtal membrane closing up the internal os uteri and the uterine openings of the Fallopian tubes. When the uterus is thus sealed, as well as filled by its fœtal contents, we must admit that another impregnation is hardly possible, because the spermatozoa cannot enter the uterus, nor can the ovum enter it from the Fallopian tubes. But this rule, like others, has its exceptions, as is abundantly proved by the fact that menstruation in the human female occasionally occurs during gestation in spite of the mucous plug.

As a rule, the ovaries are in abeyance during gestation, but these same instances of menstruation prove exceptions to this rule also. And we know of instances of mares being in heat at intervals for months after conception. I know of one case where the mare took the stallion on an average of once a month till a month before she foaled a live, well-developed colt.

Menstruation is the external proof of the escape of an ovum from the ovary, and it also proves that the os uteri is not sealed when it occurs, and there are many cases recorded of women who menstruated for some time after conception. And, on the other hand, it is asserted that women have become pregnant without ever having menstruated.

Menstruation ceases with rare exceptions when the ovaries are removed, and we know that mares, cows, and bitches rarely go in heat after ovariotomy has been performed. Hence, we may say that in those cases where the mare has been in heat after conception a Graafian vesicle has matured and an ovum has escaped from it, although, as a rule, we take it for granted that there has been no impregnation so long as the mare continues to go in heat and takes the stallion. As a rule, then, after an ovum has been fecundated, the ovaries remain in abeyance, and there are no periods of œstrum till after parturition. In cases of normal twins the ova become impregnated in the manner already explained. But occasionally a mare may conceive, go in heat again, take the stallion, and conceive the second time. Such instances come under the head of superfecundation, and there are numerous cases recorded that support the assertion, such as mares having horse and mule colts after having been served by a stallion and an ass. Bitches frequently have a litter of pups showing the different breeds of the different dogs they have taken during the same heat, but such cases are no more than ordinary conceptions, as the bitch is a multiparous animal, and the multiple ova that escape at one heat are as liable to be fecundated by the spermatozoa of one dog as another when she is allowed to be indiscriminate in her desires.

There are many cases recorded of black women having twins, one child being black, the other a mulatto, after admitted cohabitation with black and white men. There are also cases reported of white women having black and white children at the same birth. The *Medical Record* of April 27, 1895, contains the following: "Amalgamated Twins.—Dr. F. B. Heisordt (*Journal of the American Medical Association*) reports the birth of twins, the mother being a light-haired German woman and the father a negro; one of the twins was white and one black." No other particulars are given, but we can draw our own conclusions. Those opposed to the doctrine of superfecundation endeavor to explain such occurrences in women on the theory that one child takes after the father and the other after the mother, but such nonsense will not apply to the cases of mares having horse and mule colts.

(To be concluded.)

